# Examining Diagnostic Efficacy: GeneXpert Versus Traditional Staining Techniques With Culture in the Diagnosis of Tuberculosis

**DOI:** 10.7759/cureus.63641

**Published:** 2024-07-02

**Authors:** Lavanya Balaji, Lavanya Ramanan, Manivannan Nandhagopal, Jayakumar Subramaniam

**Affiliations:** 1 Department of Microbiology, Saveetha Medical College and Hospital, Saveetha Institute of Medical and Technical Sciences, Chennai, IND

**Keywords:** ziehl- neelsen (zn) stain, auramine -o stain, genexpert, tuberculosis, diagnostic techniques

## Abstract

Introduction

The tuberculosis (TB) diagnosis involves various methods, such as microscopic examination, culture-based methods, molecular techniques, chest X-rays, serological tests, and interferon-gamma release assays. These methods help identify and confirm TB and its resistance to rifampicin, balancing speed and accuracy for prompt treatment initiation and effective disease management.

Aims and objectives

To assess the diagnostic accuracy of GeneXpert, Ziehl-Neelsen staining, and fluorescence staining compared to culture media in TB-suspected patients.

Materials and methods

We analysed 416 patient samples for TB over one year using GeneXpert, Ziehl-Neelsen staining, fluorescence staining, and Löwenstein-Jensen (LJ) medium. Only samples with a suspicion of TB were included in the study. The samples received without clinical history and requests for all four tests were excluded.

Results

A total of 416 patient samples were categorised into pulmonary and extrapulmonary samples. GeneXpert detected 62 positive cases for TB, out of which 53 were rifampicin-sensitive, seven were rifampicin-indeterminate, and two were rifampicin-resistant. The indeterminate samples were further evaluated using the line probe assay (LPA), of which six were rifampicin-sensitive, and one was rifampicin-resistant. Fluorescent staining detected 44 cases, Ziehl-Neelsen staining detected 40 cases, and LJ culture medium detected 65 cases.

Conclusion

GeneXpert is superior to staining methods for detecting TB. GeneXpert, combined with microscopy and culture, can enhance TB and multi-drug resistant tuberculosis (MDR-TB) detection and aid in early treatment initiation.

## Introduction

Tuberculosis (TB) presents a significant global health challenge, with an estimated 10 million new cases and 1.3 million deaths each year [1]. A timely and accurate diagnosis is essential for effectively tackling this infectious disease. Over time, various diagnostic methods have been developed and employed in the fight against TB. The evolution of TB diagnosis has progressed from traditional approaches to more advanced techniques [2]. Initially, acid-fast staining of sputum samples was the primary diagnostic method. However, this method had limited sensitivity, particularly in cases of paucibacillary disease. Introducing culture-based methods, such as solid and liquid media cultures, enhanced sensitivity but prolonged the turnaround time, resulting in delays in treatment initiation [3]. Studies assessing TB diagnostic methods typically use reference standards like culture or clinical follow-up to establish individuals' true disease status. Sensitivity measures a test's ability to accurately identify individuals with TB, while specificity assesses its ability to correctly identify those without TB. For example, the GeneXpert assay has shown high sensitivity and specificity in detecting TB and rifampicin resistance, making it a valuable tool in TB diagnosis [4]. In high-burden settings, turnaround time is a crucial factor, as delays in diagnosis can lead to increased transmission and poor treatment outcomes. Rapid molecular tests like GeneXpert offer advantages by providing results within hours, in contrast to culture-based methods, which take six to seven weeks [5]. Cost-effectiveness analysis is crucial for evaluating the economic impact of diagnostic methods, taking into account factors such as equipment, consumables, and personnel training costs. Although molecular tests like GeneXpert may have higher initial costs, their ability to expedite diagnosis and reduce transmission can result in long-term cost savings [6]. This study assesses the efficacy of GeneXpert compared to Ziehl-Neelsen and fluorescence staining techniques with Löwenstein-Jensen (LJ) culture for diagnosing tuberculosis.

## Materials and methods

A cross-sectional comparative study was conducted at Saveetha Medical College and Hospital (SMCH) over one year, from December 2022 to December 2023. The study analysed 416 patient samples suspected of tuberculosis. The samples comprised the pulmonary group, which included sputum, and the extrapulmonary group, which included broncho-alveolar lavage, cerebrospinal fluid, tissue, pleural fluid, urine, lymph node, and pus. The samples were examined using several diagnostic tests, including Ziehl-Neelsen (ZN) and auramine rhodamine/fluorescent staining, GeneXpert (Cepheid Inc., Sunnyvale, CA, USA), and culture with Löwenstein-Jensen medium. The staining procedures for ZN and auramine-rhodamine staining were performed only after quality control (QC) was passed.

Inclusion criteria

Patients suspected of TB with the presence of various symptoms, such as cough, weight loss, fatigue, hemoptysis, and loss of appetite, were included in the study.

Exclusion criteria

Salivary samples, biopsy samples sent in formalin bottles and samples without requests for all four tests (Ziehl-Neelsen staining, fluorescent staining, GeneXpert, and LJ culture) were excluded from the study.

Sample collection and processing

The samples were collected in sterile, leak-proof containers according to the guidelines established by the National Tuberculosis Elimination Programme (NTEP) [7]. They were divided into two parts: one was used for GeneXpert and the other for direct microscopy and culture. Smears were subjected to Ziehl-Neelsen (ZN) staining and auramine-rhodamine (AR) staining. The decontamination and concentration procedures were performed using modified Petroff’s method and then inoculated on Löwenstein-Jensen (LJ) medium slopes [8].

Interpretation

**Figure 1:**
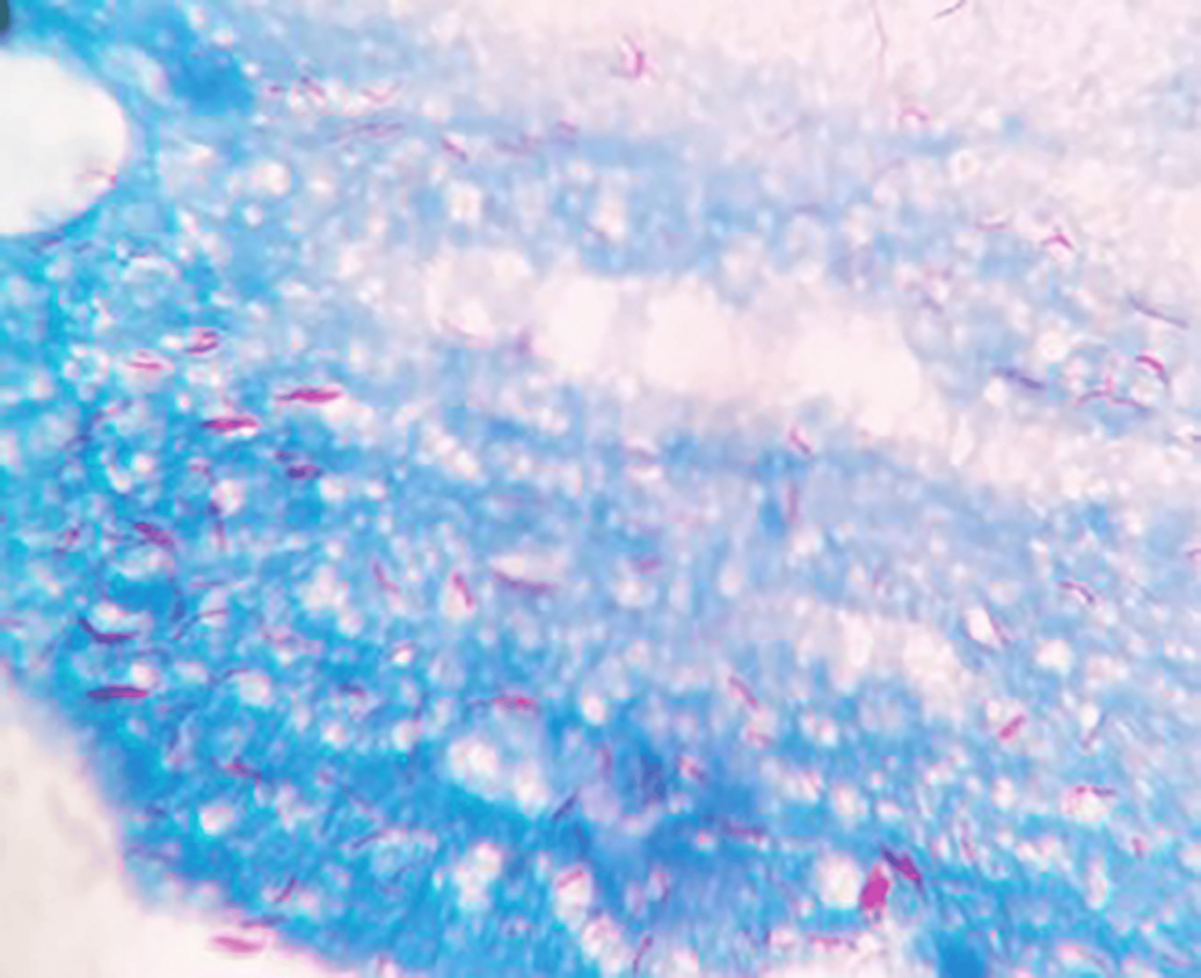
Ziehl-Neelsen (ZN) staining showing as red, curved beaded bacilli.

Acid-fast bacilli (AFB) were observed as red, curved beaded bacilli when assessed under oil immersion (X 100) as shown in Figure 1.

**Figure 2:**
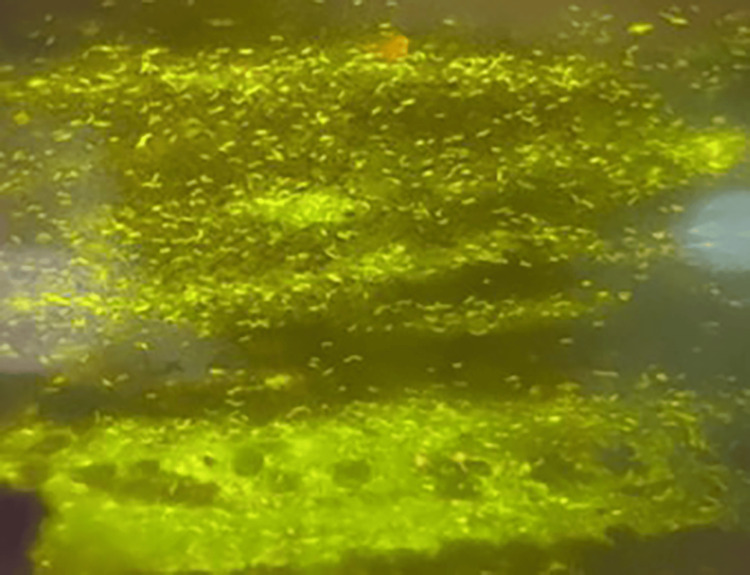
Auramine-rhodamine staining showing the tubercle bacilli appearing bright, brilliant greenish-yellow against a dark background

The smears were graded according to the NTEP guidelines for ZN staining [7]. When using auramine-rhodamine/fluorescent staining, the tubercle bacilli appeared bright and brilliant greenish-yellow against a dark background in 40 X under a fluorescent field [9] (Figure 2).

The grading was conducted in accordance with NTEP guidelines for fluorescent staining. The GeneXpert assay was performed as per the manufacturer's instructions. The cartridge was inserted into the GeneXpert module, and the results for the presence of *Mycobacterium tuberculosis* (MTB) were interpreted as either MTB detected or MTB not detected, with rifampicin resistance detected or not detected after two hours [10].

Analysis

The data were collated into a master chart using Microsoft Excel (version 2021, Microsoft Office; Microsoft, Redmond, WA) and analysed for correlation. Sensitivity, specificity, positive predictive value (PPV), and negative predictive value (NPV) were determined to diagnose TB through AFB smear microscopy and GeneXpert, with MTB culture serving as the gold standard. Samples that tested positive and negative for culture were used as a reference to determine true-positives and true-negatives. False-positives were identified as samples that tested negative for culture but positive for GeneXpert, while false-negatives were samples that tested negative for GeneXpert but positive for culture. The categorical data was computed as percentages, and the chi-square test (χ^2^ test) was performed to look for statistical significance as appropriate. A significance level of p<0.05 was considered statistically significant. 

## Results

**Figure 3:**
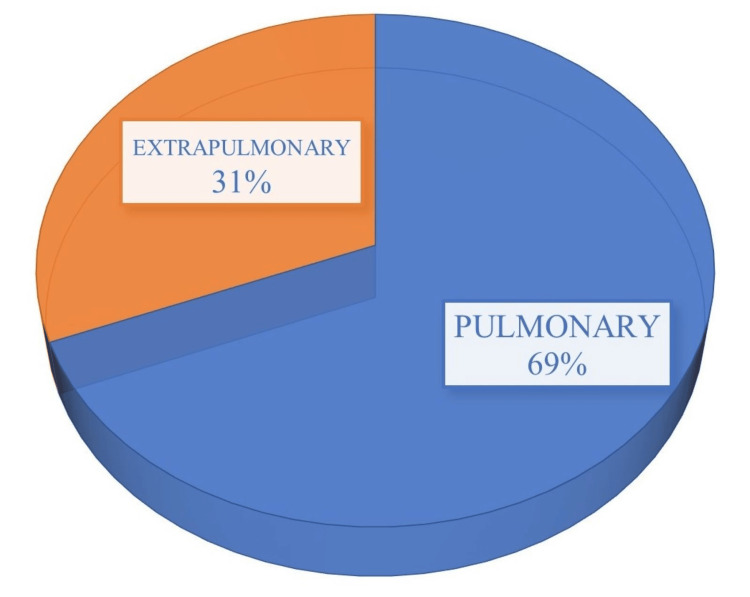
Sample-wise distribution of suspected patients with tuberculosis. The data has been represented in %.

Our study included the analysis of 416 samples categorised into pulmonary (69%, n=287) and extrapulmonary (31%, n=129). The extrapulmonary group comprised samples such as broncho-alveolar lavage, cerebrospinal fluid, tissue, pleural fluid, urine, lymph node, and pus, as depicted in Figure 3.

 

 

**Figure 4:**
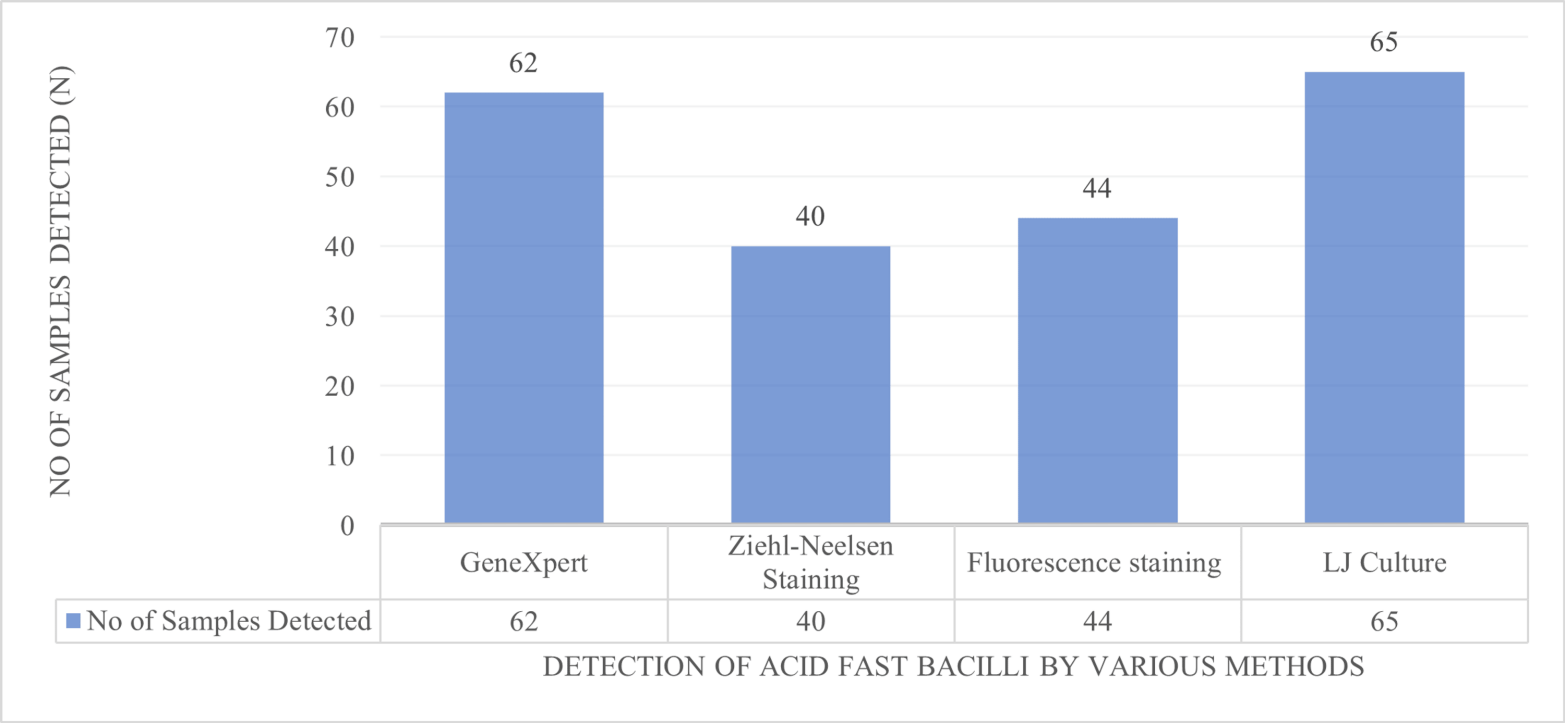
Number of positive cases detected by GeneXpert, Ziehl-Neelsen staining, fluorescence staining, and LJ culture. LJ: Löwenstein-Jensen

The majority of the participants in the study were females (n=219) compared to males (n=197). Most of the individuals tested for tuberculosis were aged between 21 and 40 years. The collected samples were analysed by different diagnostic methods, including Zeihl-Neelsen staining, auramine-rhodamine staining, GeneXpert, and culture with LJ medium. A total of 416 samples were analysed using these methods. The Ziehl-Neelsen (ZN) staining method detected 40 cases (9.61%) as positive for acid-fast bacilli while the remaining 376 cases (90.39%) tested negative. The auramine-rhodamine fluorescent staining method identified 44 cases (10.57%) as AFB positive and the remaining 372 cases (89.42%) tested negative. Culture testing with LJ medium revealed that 65 samples (15.62%) were culture-positive, with the remaining (84.38%) showing culture-negative results. The LJ culture results were regarded as the gold standard, with positive cultures being deemed true-positives and negative cultures being deemed true-negatives. The GeneXpert test detected 62 positive cases (14.90%), with 53 cases (85.48%) found to be rifampicin-sensitive, seven cases (11.2%) as rifampicin-indeterminate, and two cases (3.22%) as rifampicin-resistant. Further testing of the seven rifampicin-indeterminate samples using the line probe assay showed six cases as rifampicin-sensitive and one as rifampicin-resistant as shown in Figure 4.

**Table 1:** Comparison of various staining techniques with GeneXpert. The data has been represented in n (%) and the p value is calculated using the Chi-square (X^2^) test. *p-value is 0.040. p-value <0.05 is considered statistically significant.

Diagnostic techniques	ZN staining, n (%)	Auramine-rhodamine staining, n (%)	GeneXpert, n (%)	p-value
Positive	40 (9.61 %)	44 (10.57 %)	62 (14.90 %)	0.040*
Negative	376 (90.38 %)	372 (89.42%)	354 (85.09 %)	

A statistical comparison of diagnostic techniques including Ziehl-Neelsen staining, auramine-rhodamine staining, and GeneXpert revealed significant differences in their efficacy. The p-value of 0.040 (p<0.05) indicates statistical significance, confirming GeneXpert's superiority in the detection of acid-fast bacilli compared to staining techniques. This underscores the importance of utilising advanced molecular techniques like GeneXpert for an accurate diagnosis of tuberculosis, particularly when compared to traditional staining methods.

Distribution of cases detected by GeneXpert

**Figure 5:**
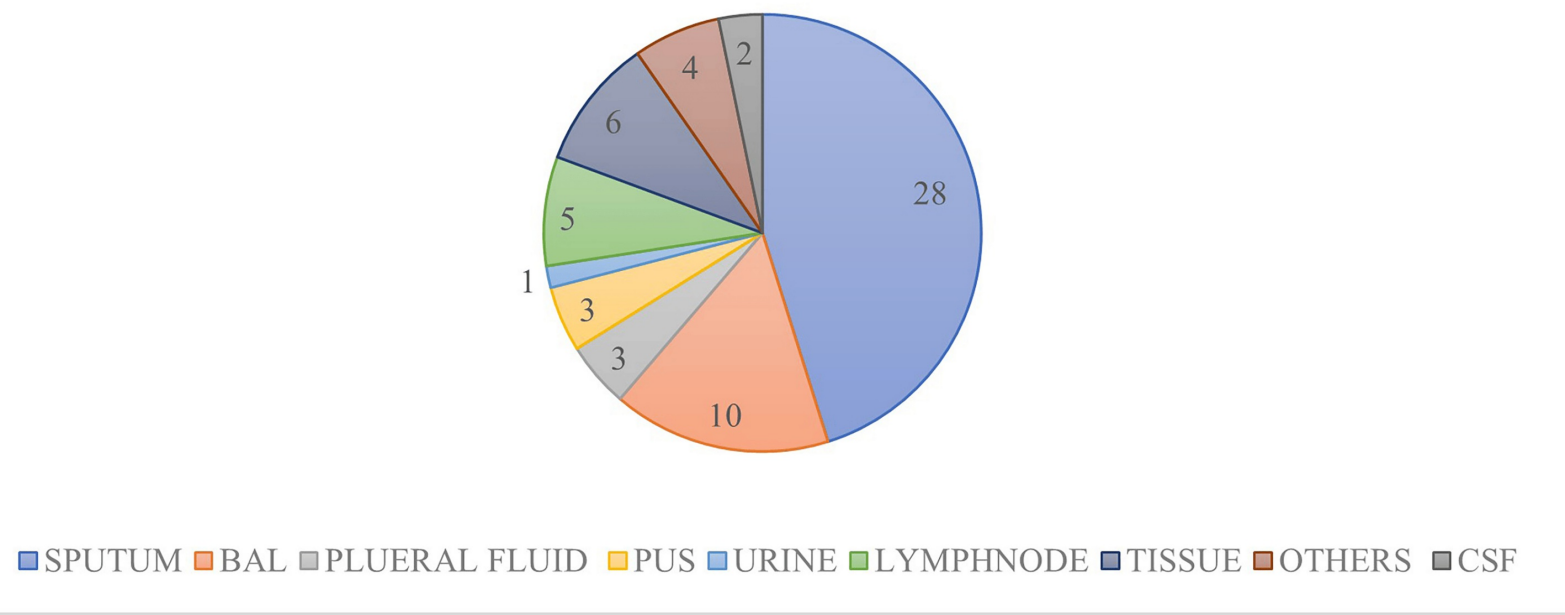
Sample-wise distribution of cases detected as positive by GeneXpert The numbers in the figure represent the number of positive cases detected by GeneXpert.

Of 62 positive cases detected by GeneXpert, the positivity rate was 54.83% (n=34) in the extrapulmonary group and 45.16 % (n=28) in the pulmonary group. The pulmonary group consisted of only sputum samples (n=28), while the extrapulmonary group had samples like broncho-alveolar lavage, cerebrospinal fluid, tissue, pleural fluid, urine, lymph node, and pus. Their distribution is displayed in Figure 5.

**Figure 6:**
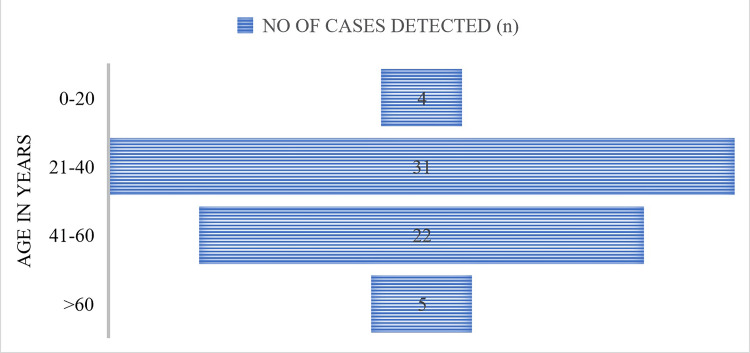
Age-wise distribution of cases detected by GeneXpert.

**Figure 7:**
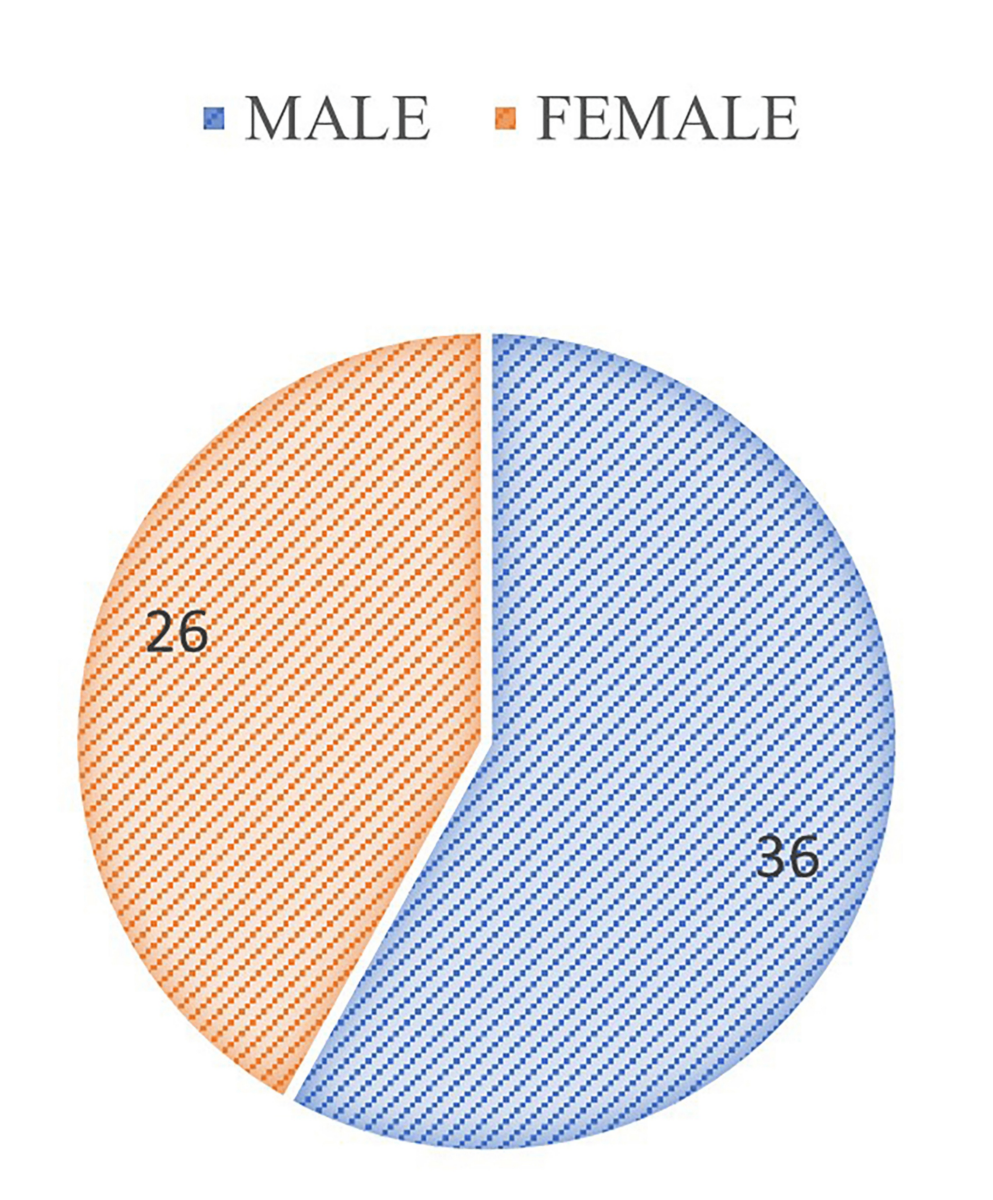
Gender-wise distribution of cases detected by GeneXpert

The highest number of detected cases were in the 21-40 age group, followed by the 41-60 age group. The prevalence of positivity was higher in males at 58% (n=36) than in females at 42% (n=26), as shown in Figures 6, 7.

In this study, the GeneXpert assay emerged as the most sensitive diagnostic tool, demonstrating an impressive sensitivity of 98.4% and a specificity of 100%. This indicates that the GeneXpert accurately identified 98.4% of true-positive cases and consistently ruled out tuberculosis in all true-negative cases. Following closely behind, fluorescent staining showed a sensitivity of 88.01% and a specificity of 100%, highlighting its reliability in detecting tuberculosis cases, albeit with slightly lower sensitivity compared to GeneXpert. Zeihl-Neelsen staining, while still effective, exhibited a sensitivity of 83.32% and maintained a specificity of 100%. Although Zeihl-Neelsen staining demonstrated slightly lower sensitivity than GeneXpert and fluorescent staining, its consistent specificity suggests its usefulness in confirming tuberculosis cases.

The gold standard, Löwenstein-Jensen medium, demonstrated 100% sensitivity and specificity, correctly identifying all true-positive cases without any false-positives and accurately ruling out tuberculosis in all true-negative cases as seen in Table 2. This emphasizes its reliability as a reference standard for diagnosing tuberculosis.

**Table 2:** Comparison of various staining techniques with GeneXpert LJ: Löwenstein–Jensen The data has been represented in %.

Diagnostic techniques	Sensitivity (%)	Specificity (%)	Positive predictive value (%)	Negative predictive value (%)
Ziehl-Neelsen staining	83.32%	100%	100%	97.90%
Fluorescent staining	88.01%	100%	100%	98.40%
GeneXpert	98.40%	100%	100%	100%
LJ medium	100%	100%	100%	100%

## Discussion

Effective testing is crucial in the TB care pathway. Proper microbiological detection is vital since it enables an accurate diagnosis and the initiation of the most effective treatment regimen at the earliest possible stage. People diagnosed with TB without bacteriological confirmation are considered "clinically diagnosed" cases [11]. Bacterial confirmation is necessary to test for resistance to anti-TB drugs. Rapid molecular tests, phenotypic susceptibility testing, or genetic sequencing (at reference-level laboratories) can all be used for this purpose.

In 2022, 7.5 million people globally were newly diagnosed with TB and reported as TB cases, with 6.2 million (83%) of them having pulmonary TB. Between 2018 and 2021, the percentage of people diagnosed with TB based on bacteriological confirmation increased from 55% to 63% worldwide and remained at 63% in 2022 [12]. To manage TB, a laboratory-based diagnostic approach relies on initial microscopic examination and clinical confirmations, with newer advanced diagnostic tools available, such as genotypic assays (line probe assay, cartridge-based nucleic acid amplification test, loop-mediated isothermal amplification) for rapid molecular testing and culture methods (liquid culture media) with standard drug susceptibility testing assays. GeneXpert MTB/RIF analysis is nowadays the preferred choice due to its rapid results and high sensitivity [13].

The study compared four methods for detecting acid-fast bacilli in the diagnosis of pulmonary TB. GeneXpert showed the highest detection rate (14.90%), followed by fluorescent staining (10.57%) and ZN staining (9.67%). Previous studies have also favoured auramine-rhodamine staining over Zeihl-Neelsen staining [14]. While fluorescent microscopy is more sensitive, its disadvantage lies in fading fluorescence and cost, necessitating reading within 24 hours. However, Zeihl-Neelsen staining is globally preferred due to its simplicity and cost-effectiveness. World Health Organization (WHO) recommends light-emitting diode (LED) microscopy as an alternative to conventional fluorescence and Zeihl-Neelsen microscopy [15]. GeneXpert exhibited higher sensitivity and specificity (100%) than staining techniques, detecting 20 additional MTB-positive cases categorised as smear-negative in our study. Studies by Hamal et al. [16] and Umair et al. [17] have also been concordant with the present findings. Among staining techniques, fluorescent staining was more sensitive, followed by Zeihl-Neelsen staining, favoured by the findings of a study done by Golia et al. [18]. Studies by Aggarwal et al. [19] Ondimu et al. [20], Nakate et al. [21] , and Dzodanu et al. [22] also supported GeneXpert's superiority in sensitivity. GeneXpert's sensitivity advantage over AFB smear microscopy is attributed to its lower detection threshold (131 bacilli/ml vs. 5,000 bacilli/ml) [23]. The gold standard for TB detection remains the culture method. In our study, culture with LJ medium demonstrated 100% sensitivity and specificity for TB detection.

Limitations of the study

Notably, three cases undetected by GeneXpert were identified through growth in the LJ medium. The negative GeneXpert results could be attributed to the presence of atypical *Mycobacterium tuberculosis* or non-tuberculous mycobacterium, or a very low/trace bacilli load. While GeneXpert is costlier, its sensitivity makes it preferable to avoid prolonged morbidity and costs associated with missed diagnoses. The GeneXpert system can only detect a bacilli concentration of 131 per ml or higher in a given sample. For extrapulmonary samples such as pus and tissues, where the concentration of *Mycobacterium tuberculosis* is in a trace amount, low, or very low, the system often cannot detect rifampicin resistance. In such cases, the result is typically interpreted as intermediate. In conclusion, GeneXpert's sensitivity outperforms staining techniques, justifying its recommendations where available to enhance TB diagnosis efficacy.

## Conclusions

GeneXpert offers increased sensitivity and delivery of rapid results over the staining techniques and culture in diagnosing TB. Unlike LJ culture, which takes days for results and cannot detect rifampicin resistance simultaneously, GeneXpert diagnoses tuberculosis irrespective of acid-fast bacilli smear status and identifies rifampicin resistance in tertiary care setup, which is crucial in the case of multi-drug-resistant and HIV-associated TB. However, caution is advised when interpreting reports of positive culture and negative polymerase chain reaction (PCR) results, which necessitates a correlation between clinical and treatment history.
